# Comment on “survival of patients with 2014 FIGO stage IIIC high-grade serous ovarian cancer treated with platinum-based adjuvant chemotherapy”

**DOI:** 10.55730/1300-0144.6173

**Published:** 2025-11-13

**Authors:** Abdullah SAAD, Syeda Abeeha HASAN, Syeda Akifa SAJID

**Affiliations:** Division of MBBS, Department of Internal Medicine, Faculty of Medicine, Sindh Medical College, Jinnah Sindh Medical University, Karachi, Pakistan

**Dear Editor**,

We read with great interest the article by Ünsal et al., which examined survival outcomes and prognostic factors in patients with FIGO stage IIIC high-grade serous ovarian cancer (HGSOC) treated with platinum-based adjuvant chemotherapy [1]. These findings contribute to a critical domain of gynecologic oncology, where individualized treatment planning remains challenging. However, several aspects require clarification to improve the transparency and interpretation of the results.

First, although the authors reported a well-defined and homogeneous cohort, the absence of detailed surgical selection criteria—such as performance status, tumor burden scores, or imaging-based assessment—makes it difficult to determine whether treatment decisions were patient- or surgeon-driven. This omission raises concerns about potential selection bias [2].

Second, the study did not include critical intraoperative parameters, such as surgical complexity, case distribution among surgeons, or institutional surgical volume. These variables significantly affect cytoreductive outcomes and survival in patients with HGSOC. The absence of these factors introduces residual confounding [2].

Third, the study lacks information regarding chemotherapy adherence. The authors did not specify whether patients completed chemotherapy, underwent dose reductions, or discontinued treatment early. These critical data points directly influence survival outcomes. A recent retrospective Thai cohort study (n = 757) found that older age, comorbidities, and the absence of surgery independently reduced chemotherapy completion, with completion rates of 84.3% in older versus 92.6% in younger patients [3].

Fourth, the statistical methodology used in the survival analysis lacks essential validation. Although Cox proportional hazards modeling was applied, the authors did not report whether the proportional hazards (PH) assumption was tested. This represents a major concern, as recent analyses have demonstrated that violations of the PH assumption are common and may invalidate the interpretation of hazard ratios. For instance, a 2022 analysis of oncology trials reported PH violations in nearly 24% of phase III studies, yet only a small fraction implemented corrective modelling [4].

Fifth, although the study identified high-volume ascites as an independent predictor of disease recurrence, the clinical relevance of this finding remains insufficiently explored. Ascites volume likely reflects advanced peritoneal involvement and aggressive tumor biology. In a 2023 multicenter study, ascites volumes ≥2 L were associated with lower optimal cytoreduction rates and significantly poorer progression-free and overall survival [5]. Without prospective validation or biological correlation, the prognostic significance of ascites remains speculative.

Sixth, the handling of missing data, censoring mechanisms, and loss to follow-up was not adequately described. These are fundamental components of survival analysis, and their omission may introduce substantial bias or result in an overestimation of survival outcomes [6].

Finally, a major limitation of this study is the absence of molecular profiling. Breast cancer gene mutations and homologous recombination deficiency status are well-established prognostic and predictive biomarkers for HGSOC. Their absence from the analysis limits the generalizability of the study in the era of molecularly guided therapy [7].

In conclusion, the authors present valuable observational data supporting the benefits of maximal cytoreduction in advanced HGSOC. However, prospective validation is required. Future studies should incorporate treatment adherence metrics, surgical complexity indices, and molecular markers to improve clinical applicability and accuracy.
